# Comparative analysis of the microbiotas and physicochemical properties inside and outside medium-temperature *Daqu* during the fermentation and storage

**DOI:** 10.3389/fmicb.2022.934696

**Published:** 2022-07-27

**Authors:** Xiaoge Hou, Ming Hui, Zhongke Sun, Xuesi Li, Xin Shi, Ran Xiao, Junfei Wang, Chunmei Pan, Ruifang Li

**Affiliations:** ^1^School of Biological Engineering, Henan University of Technology, Zhengzhou, China; ^2^School of Food and Biological Engineering, Henan University of Animal Husbandry and Economy, Zhengzhou, China; ^3^College of Science, Henan University of Technology, Zhengzhou, China; ^4^Key Laboratory of Functional Molecules for Biomedical Research, Henan University of Technology, Zhengzhou, China

**Keywords:** MT-*Daqu*, microbial community, physicochemical indices, volatile compounds, fermentation, mature

## Abstract

Medium-temperature *Daqu* (MT-*Daqu*), a saccharification-fermentation agent and aroma-producing agent, is used to produce Chinese strong-flavor *Baijiu*. Many related studies have been published; however, less is known about microbial community and quality properties inside and outside the MT-*Daqu* from fermentation to storage. Here, along with determining the physicochemical index, the microbial community of MT-*Daqu* was investigated using both culture-dependent and culture-independent methods during 31 days of fermentation and 4 months of storage. Volatile compounds of mature MT-*Daqu* were analyzed using headspace solid-phase microextraction (HS-SPME) combined with gas chromatography-mass spectrometry (GC–MS). The results indicated obvious variation in the microbial community due to the changes in environmental conditions, and the physicochemical indices shifted from fluctuations in the fermentation period to relative stability after storage for 3 months. Moreover, the microbial counts and physicochemical indices of the inner layers of MT-*Daqu* differed from those of the outer layers. The dominant communities, including the bacterial phyla *Firmicutes*, *Proteobacteria*, and *Actinobacteria* and the fungal phyla *Ascomycota* and *Mucoromycota*, showed different abundances in the two parts of the mature MT-*Daqu*, and different microbial communities were enriched in both parts. Additionally, pyrazines and alcohols were the most abundant volatile aroma compounds in the mature MT-*Daqu*.

## Graphical Abstract

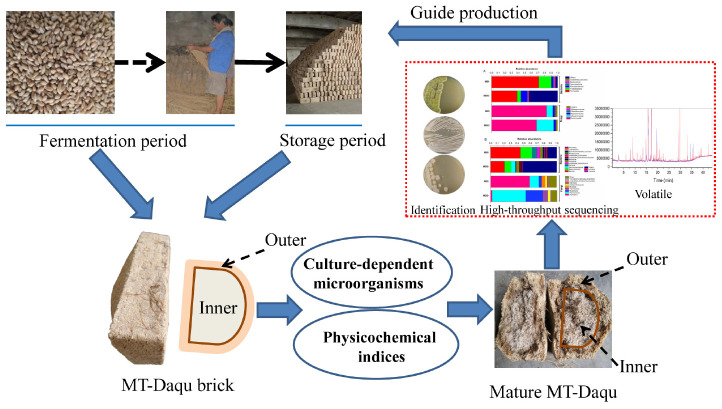

## Introduction

*Baijiu* is one of the six well-known distillates worldwide (Jin et al., 2017; He et al., 2019), and *Daqu* was used as a saccharification and fermentation agent and aroma-producing agent for *Baijiu* production (Wang et al., 2017). Thus, *Daqu* constituents exert a direct effect on the final distilled *Baijiu*. Due to the abundant accumulation of microorganisms, enzymes and flavor compounds are formed during an open spontaneous solid-state fermentation process (Cai et al., 2021; Deng et al., 2021). According to the highest fermenting temperature, *Daqu* is mainly classified into three types: high-temperature *Daqu* (60–70°C), medium-temperature *Daqu* (50–60°C), and low-temperature *Daqu* (40–50°C) (Deng et al., 2020; Cai et al., 2021; Hu et al., 2021; Wang et al., 2021). These three types of *Daqu* have been employed to produce *Jiang*-flavor, strong-flavor, and light-flavor *Baijiu*, which are the three most popular *Baijiu* products on the Chinese market (Hu et al., 2021; Zhang H. et al., 2021).

Strong-flavor *Baijiu*, with ethyl caproate as the main aroma, has the characteristics of a fragrant flavor, soft mouthfeel, and long-lasting aftertaste and is the most famous type of *Baijiu*, with an annual output of more than 70% of all Chinese liquor (Xu et al., 2017; Zou et al., 2018; Deng et al., 2021). The generation of the flavor of strong-flavor *Baijiu* is closely linked to MT-*Daqu*. As a saccharifying starter for 1000 of years and as raw material, MT-*Daqu* is combined with approximately 20% raw grains for strong-flavor *Baijiu* fermentation (Xu et al., 2017; Zou et al., 2018). MT-*Daqu* is produced by solid fermentation using a wheat or wheat mixture as raw materials, and the process involves three phases: material mixing and shaping, spontaneous solid-state fermenting, and maturing (Supplementary Figure 1; Zou et al., 2018). During MT-*Daqu* production, the fermentation phase is crucial and proceeds through five stages, namely, the *Anqu*, *Peijun*, *Dinghuo*, *Huanluo*, and *Dalong* phases (Yan et al., 2019). As a result, numerous microorganisms, such as *Bacillus*, *Acetobacter*, *Pseudomonas*, *Weissella*, *Alternaria*, *Aspergillus, Mucor*, *Saccharomyces*, and *Hansenula*, inhabited the fermentation broth, and enzymes, such as liquefaction enzymes, saccharifying enzymes, proteases, and esterifying enzymes, accumulated through microbial metabolism. Flavor compounds, such as organic acids, alcohols, esters, aldehydes, and ketones, contributing to the generation of flavor of *Baijiu* are also enriched in this period (Yan et al., 2013a,^2019^; Liu et al., 2018; Deng et al., 2021). Additionally, fresh *Daqu* must mature for 3–6 months to exclude undesirable microorganisms and balance metabolic compounds (Fan et al., 2019; Yang et al., 2021). Only mature *Daqu* can be used for *Baijiu* brewing. Therefore, understanding the rule of microbial succession and metabolism in the MT-*Daqu* production process might contribute to the stability and quality of *Daqu*.

However, the microbial composition and physicochemical indices in *Daqu* undergo continuous succession during production due to environmental factors (Fu et al., 2021). Meanwhile, different microbiotas and physicochemical properties have been observed in different parts of the same *Daqu* brick due to the difficulty in transferring heat, water, and oxygen (Jin et al., 2019; Chen et al., 2020; Zhang Y. et al., 2021). The aforementioned results all affect the quality of *Daqu*. At present, the criteria that are accepted by *Baijiu* industry have clearly specified the organoleptic requirement and predominant physicochemical index requirement for a fermentation MT-*Daqu* that is mature (People’s Republic of China Light Industry Professional Standard., 2011b), QB/T 4259-2011, 2011; (Shangdong Province Local Standard, 2009, in China, DB37/T 1231-2009, 2009). The microorganism and the physicochemical property indices inside and outside the strong flavor *Daqu* are not included in the industrial criteria. Therefore, analyzing the microbiotas and physicochemical indices inside and outside the *Daqu* during production might provide a scientific theory for establishing a relatively comprehensive method to evaluate the quality and control the process of *Daqu*. Some studies on these have been performed (Jin et al., 2019; Chen et al., 2020; Zhang Y. et al., 2021). Jin et al. (2019) revealed that the dominant microbes and volatile flavors on the surface and the core of soy-flavor *Daqu* had different distribution characteristics and found that microbial interactions were stronger in the core than on the surface (Jin et al., 2019). Thereafter, Zhang Y. et al. (2021) explored the microbiota, enzymes, and metabolites in different parts of fresh and mature soy-flavor *Daqu*, and the dominant enzymes varied significantly and identified the predominant microbial community migrated between the two parts of *Daqu* during production (Zhang Y. et al., 2021). In addition to *Jiang*-flavor *Daqu*, studies on differences in microbiota and physicochemical indices between surface and central parts of special-flavor *Baijiu Daqu* were reported, and the microbial distribution and physicochemical indices in the two parts were significantly different (Chen et al., 2020). These findings potentially provide a theoretical basis for microbial resources and quality control of *Daqu* (Wang et al., 2021). However, Chinese flavor *Baijiu* brewing enterprises are widely distributed over South China and North China and have their own unique ecological environments and diverse regulations for the production of *Daqu*. Therefore, a comprehensive understanding of the mechanisms underlying the composition of *Daqu* might contribute to better control of the *Daqu* process and standardize local *Baijiu* production (Xu et al., 2017; Liu et al., 2018). Currently, studies that examine different parts or different types of *Daqu* mainly focus on high-temperature or low-temperature *Daqu* (Chen et al., 2020; Cai et al., 2021; Wang et al., 2021; Zhang Y. et al., 2021), and a comprehensive study of the microbial compositions and the quality characteristics of different parts of MT-*Daqu* have not yet been conducted.

In this study, the bacterial and fungal communities in different parts of strong-flavor *Daqu* from the fermentation period to the mature period were investigated using a culture-dependent method, and the changes in the physicochemical properties throughout the production process were also explored. Based on these results, the maturity period of MT-*Daqu* was evaluated, and the analysis of microbial composition, physicochemical indices, and volatile compounds of different parts of mature MT-*Daqu* using the Illumina MiSeq platform and headspace solid-phase microextraction (HS-SPME)-GC–MS was the main focus. This study provides guidance for the determination of the mature stage, for the process control of MT-*Daqu*, and for improving the quality of strong-flavor *Baijiu*.

## Materials and methods

### Production of medium-temperature-*Daqu* and sample collection

Medium-temperature-*Daqu* samples were obtained from Songhe Co., Ltd., in Henan, China (longitude 115.48°E and latitude 33.86°N), a typical distillery that produces strong-flavor *Baijiu* in Henan Province. MT-*Daqu* was produced by traditional solid-state fermentation in summer (Supplementary Figure 1). The same batch of MT-*Daqu* bricks was removed from the fermentation room on days 1, 5, 9, 13, 17, 21, 26, and 31 and from the storage room at 0.5, 1, 1.5, 2, 3, and 4 months. The *Daqu* samples were collected as described in the previous reports (Yan et al., 2019). A total of three *Daqu* bricks were randomly selected from the top, middle, and bottom of stacked layers. Subsequently, each *Daqu* brick was divided into two parts based on the fire-circle strategy (Wang et al., 2020): the 0.5–2-cm thick outer layer (named MDO) and the inner layer of the remaining part (named MDI). Then, the same parts (equal weight) of each *Daqu* brick were mixed into one sample and separated into two parts for subsequent testing. Samples were collected during two parallel batches of MT-*Daqu* production, and four biological replicates of each sample were obtained. Approximately 50 g of the sample was immediately brought to the laboratory to analyze the microbial community, 100 g of sample was stored at –20°C for the analysis of physicochemical indices and volatile compounds, and 50 g of sample was sealed in sterile bags and stored at –80°C for Illumina MiSeq sequencing.

### Analysis of environmental factors and physicochemical indices

During the MT-*Daqu* fermentation period, the temperature and humidity in the fermentation room and the interior of *Daqu* bricks were measured using thermometers and hygrometers, respectively (Li et al., 2016). The physicochemical indices were determined according to the industrial general standard methods with a slight modification (People’s Republic of China Light Industry Professional Standard., 2011a), QB/T 4257-2011, 2011; Shangdong Province Local Standard in China, DB37/T (1231-2009). Moisture (g/g,%) was measured as the weight loss of the samplers after drying at 105°C for 3–5 h until constant weight. The total acidity (mmol/10 g) was determined by titrating with 0.1 M NaOH to pH 8.2. Liquefying activity (g/g● h) was determined by the reaction time (minutes) with a mixture of iodine solution and starch solution. Saccharifying activity (mg/g●h) was measured using Fehling’s solution titration method (Xiao et al., 2017). The esterifying activity (mg/g●100 h) was determined using the saponification method and reported as the amount of ethyl caproate synthesized from caproic acid and ethanol per 100 h by 1 g of dried *Daqu*. Fermenting activity (gCO_2_/g●72 h) was determined by the weight loss of sorghum saccharifying solution incubated with 0.5 g of *Daqu*, and it was reported as the amount of CO_2_ released in 72 h by 1 g dried *Daqu*.

### Culture-dependent analysis of representative microorganisms in medium-temperature-*Daqu*

Microbial enumeration using culture-dependent methods has described in the previous study (Hu et al., 2017).

About 10 g of *Daqu* sample was mixed with 90 ml of sterile saline (0.85% w/v sodium chloride) in a-250 ml sterile conical flask and shaken at 25°C for 30 min at 120 rpm to obtain homogenized samples. After allowing the mixture to stand for a few minutes, the supernatant was diluted 10-folds. Next, 0.2 ml of the diluted suspension was spread in triplicate on the surface of plate count agar medium (Oxoid CM0325) and cultured at 37°C for 24 h to enumerate total aerobic bacteria and spread on potato dextrose agar medium (Oxoid CM0139) and Czapek Dox medium containing 0.1 g/L ampicillin and cultured at 30°C for 3 days to enumerate yeasts and molds (Wang et al., 2018). For the estimation of spore-forming bacteria, a 10% (w/v) sample suspension was heated at 80°C for 10 min and then spread on plate count agar medium, followed by incubation at 55°C for 72 h (Zheng et al., 2012). Colony-forming units (CFUs) per gram of fresh weight sample were calculated based on their morphological features.

Considering the application of the mature MT-*Daqu* for *Baijiu* production, the strains from the mature MT-*Daqu* were isolated and identified. A total of five to ten colonies with the square root of the total number of colonies were picked randomly from each plate for purification using the successive streak plate method (Yang et al., 2022). Genomic DNA was used as a template for PCR to amplify the bacterial 16S rDNA using the universal primer pairs 27F and 1492R, the mold 18S rDNA using the primers NS1(5′-GTAGTCATATGCTTGTCTC-3′) NS6 (5′-GCATCACAGACCTGTTATTGCCTC-3′), and the yeast internal transcribed spacer (ITS) regions using the universal primers ITS1/ITS4 (Zhang H. et al., 2021). Then, purified PCR products were sequenced by Sangon Biotech Co., Ltd. (Shanghai, China), and the sequences were submitted to GenBank for comparison with those in the NCBI GenBank database using the BLAST search tool for the identification of the strains.

### Culture-independent analysis of the microbial community in mature medium-temperature-*Daqu*

Mature MT-*Daqu* samples were removed for Illumina sequencing. Metagenomic DNA was extracted using the CTAB/SDS method, and DNA concentration and purity were monitored on 1% agarose gels. According to the concentration, DNA was diluted to 1 ng/μl using sterile water (Xiao et al., 2017). Variable regions V4 of the bacterial 16S rRNA gene were amplified with degenerate PCR primers: 515F (5′-GTGCCAGCMGCCGCGGTAA-3′) and 806R (5′-GGACTACHVGGGTWTCTAAT-3′). ITS1 of the ITS region was amplified with degenerate PCR primers: ITS1F (CTTGGTCATTTAGAGGAAGTAA) and ITS2R (GCTGCGTTCTTCATCGATGC). The PCR products were purified with a Qiagen Gel Extraction Kit (Qiagen, Germany), sent to Novogene Co., Ltd. (Beijing, China), and sequenced using the Illumina NovaSeq platform. The raw 16S rRNA and ITS gene sequencing reads were merged using FLASH version 1.2.7, quality-filtered using the QIIME (v1.9.1) quality control process, and the effective tags were finally obtained by removing chimera sequences using the UCHIME algorithm (UCHIME Algorithm). The sequence analysis was performed using UPARSE v7.0.1001, and sequences with ≥97% similarity were assigned to the same OTUs. Rarefaction curves were obtained by comparing the richness and evenness of OTUs among samples. A representative sequence for each OTU was screened for annotation using the Silva Database (Release 138) for bacteria and the Unite Database (Release 8.0) for fungi. The species diversity, including alpha diversity and beta diversity, was analyzed using QIIME (1.9.1) software and displayed with R software (version 2.15.3). The original sequence was deposited in the NCBI Sequence Read Archive (SRA, PRJNA815814).

### Analyses of volatile compounds in mature medium-temperature-*Daqu* using headspace solid-phase microextraction gas chromatography-mass spectrometry

Volatile compounds in mature MT-*Daqu* were analyzed using HS-SPME-GC–MS. Using a 50/30 μm DVB/CAR/PDMS fiber (Supelco, Bellefonte, PA, United States) preconditioned in the GC injection port at 250°C for 30 min, volatile extraction was performed using a published method with a minor modification (He et al., 2019). Briefly, 2.0 g of *Daqu* powder and 5 μl internal standard (2-octanol, 0.0722 g/L, Sigma-Aldrich, St. Louis, MO, United States) were placed into a 20-ml headspace vial containing 5 ml of saturated sodium chloride solution. Then, the vial was placed in a thermostatic block stirrer to equilibrate for 10 min at 50°C and subsequently extracted for 45 min with stirring at 500 r/min. After extraction, the SPME fiber was withdrawn into the needle and immediately inserted into the injection port of the Thermo Fisher Scientific GC–MS system coupled with a trace TR-WAX fused silica capillary column (30 m × 0.25 mm i.d., 0.25-μm film thickness, Thermo Fisher Scientific, United States). Ultrahigh-purity helium was used as the carrier gas at a constant flow velocity of 1 ml/min, and the temperature of the injector and detector was maintained at 250°C. Split injection (10:1) was used. The oven temperature was programmed as follows: 35°C for 2 min, 5°C/min ramp to 110°C for 5 min, and 7°C/min ramp to 230°C for 7 min. The temperatures of the interface and ion source were 250 and 240°C, respectively. The electron impact (EI) ionization energy was 70 eV. EI spectra were recorded in the 30–450 amu range in full scan acquisition mode. Volatile compounds were identified by comparison with the MS spectral database library of the National Institute for Standards and Technology (NIST 2.3, 2017) and were required to fit logically with respect to the retention time in the chromatograms. Semiquantitative determination of the compounds was conducted using a reported method (Tang H. L. et al., 2019).

### Statistical and bioinformatic analyses

The biomass of every group of microbes was determined by counting viable colonies on agar in plates with appropriately diluted samples and calculated with the formula LogCFU/g using EXCEL2016. The statistical significance of differences between the samples was determined using SPSS Statistics 16.0 software with one-way ANOVA followed by Duncan’s test, and significant differences were defined as *p* < 0.05 or *p* < 0.01. The linear relationship between predominant physiochemical indices was analyzed by calculating the Pearson correlation coefficient using SPSS 16.0 software. Principal coordinates analysis (PCoA) was applied to explore the possible differences between the samples based on Bray–Curtis distances *via* ANOSIM using R (version 3.3.1). The linear discriminant analysis (LDA) effect size (LEfSe) method was used to identify significant variations among different parts of MT-*Daqu* samples (LDA > 3.5, *p* < 0.05).

## Results

### Dynamics of environmental factors and physicochemical indices

The core temperature of MT-*Daqu* reached 60°C after 7 days, maintained 60°C for 7 days, and then decreased gradually until 24 days (Supplementary Figure 2). The relative humidity of fermentation room was close to saturation (100%) after 5 days. As shown in Table 1, the moisture of MDO significantly decreased to 14.18% in the first 9 days (*p* < 0.05), while that of MDI began to decrease from 34.20%, and the moisture in MDI was higher than that in MDO from 5 days to 75 days; then, the moisture content of both sections reached a plateau (<13%) after storage for 2 months. Similarly, the acidity of MDI was higher than that of MDO before 3 months, whereas the acidity of MDO changed slightly, but that of MDI increased sharply in the first 5 days (*p* < 0.05), peaked (2.10 mmol/10 g) on the 5th day, and then gradually decreased to a relatively stable value (1.0 mmol/10 g). In contrast, the liquefying activity and saccharifying activity of MDO were both higher than those of MDI after fermentation for 5 days (*p* < 0.05). The saccharifying activity in MDI decreased substantially from 722.50 mg/g●h to 22.50 mg/g●h in the early stage of fermentation, then increased slightly, and finally exhibited a relatively stable range from 222 to 450 mg/g●h. The saccharifying activity and liquefying activity in MDO increased to relatively stable ranges of 852 to 925 mg/g●h and 3.10 to 3.92 g/g●h after 5 days, respectively. The esterifying activity in the two parts of MT-*Daqu* exhibited a relatively high value during storage and tended to be consistent after 75 days (> 12.0 mg/g●100 h). The fermenting activity in both MDI and MDO underwent similar changes: increasing within 1–5 days, decreasing gradually within 5–31 days, and then plateauing at approximately 1.50 gCO_2_/g●72 h until the end of storage.

**TABLE 1:** Differences in physicochemical indices of samples during MT-*Daqu* production.

MT-*Daqu* sample	Phase	Time (days)	Moisture (%)	Acidity (mmol/10 g)	Liquifying activity (g/g⋅h)	Saccharifying activity (mg/g⋅h)	Esterifying activity (mg/g⋅100h)	Fermenting activity (gCO_2_/g⋅72 h)
MDI	Fermentation	1	33.85 ± 1.41^a^	0.40 ± 0.08^h^	0.25 ± 0.15^cde^	722.50 ± 10.61^a^	5.71 ± 0.22^e^	1.79 ± 0.49^ab^
		5	34.40 ± 0.00^a^	2.10 ± 0.07^a^	0.29 ± 0.03^cde^	22.50 ± 10.61^f^	5.49 ± 1.46^e^	2.02 ± 0.11^a^
		9	34.20 ± 1.13^a^	1.90 ± 0.03^a^	0.18 ± 0.10^de^	52.50 ± 3.54^f^	8.61 ± 0.98^cd^	1.75 ± 0.08^abc^
		13	29.65 ± 1.06^b^	1.40 ± 0.00^b^	0.18 ± 0.06^e^	95.00 ± 7.07^f^	8.14 ± 0.79^cd^	1.64 ± 0.19^abc^
		17	23.70 ± 1.97^c^	1.30 ± 0.14^bc^	0.45 ± 0.15^b^	260.00 ± 14.14^de^	6.78 ± 1.00^de^	1.31 ± 0.03^cd^
		21	17.98 ± 0.45^d^	1.1 ± 0.07^de^	0.50 ± 0.09^b^	335.00 ± 91.92^cd^	8.930 ± 0.80c	0.95 ± 0.01^d^
		26	16.45 ± 0.14^de^	1.00 ± 0.00^ef^	0.55 ± 0.03^b^	222.50 ± 53.03^e^	8.67 ± 1.00^cd^	0.98 ± 0.01^d^
		31	18.17 ± 0.88^d^	1.20 ± 0.00^cd^	1.33 ± 0.06^a^	440.00 ± 84.85^bc^	13.95 ± 0.50^ab^	0.95 ± 0.02^d^
	Storage	45	15.5 ± 0.50^ef^	0.90 ± 0.14^ef^	0.37 ± 0.02^bc^	287.50 ± 60.10^de^	12.45 ± 0.80^b^	1.79 ± 0.31^ab^
		60	12.68 ± 0.32^f^	1.00 ± 0.07^ef^	0.38 ± 0.07^bc^	284.00 ± 36.77^de^	13.15 ± 0.80^ab^	1.45 ± 0.10^bc^
		75	13.295 ± 0.12^f^	1.00 ± 0.07^ef^	0.20 ± 0.11^bc^	317.50 ± 24.75^de^	13.65 ± 0.06^ab^	1.62 ± 0.09^bc^
		90	12.09 ± 0.30^f^	0.90 ± 0.07^f^	0.40 ± 0.05^bc^	351.13 ± 15.73^bcd^	12.09 ± 0.04^b^	1.49 ± 0.12^bc^
		120	12.12 ± 1.24^f^	1.00 ± 0.03^ef^	0.45 ± 0.10^bc^	450.00 ± 42.43^b^	13.88 ± 0.49^ab^	1.31 ± 0.10^cd^
		150	12.13 ± 0.78^f^	0.70 ± 0.05^f^	0.50 ± 0.08^bc^	424.00 ± 50.91^bc^	14.86 ± 0.40^a^	1.54 ± 0.06b^c^
MDO	Fermentation	1	34.80 ± 0.28^a^	0.63 ± 0.09^cd^	0.20 ± 0.00^d^	725.00 ± 7.07^a^	5.91 ± 0.90^g^	2.00 ± 0.24b^c^
		5	22.50 ± 0.35^b^	0.80 ± 0.00^b^	3.10 ± 0.14^c^	855.00 ± 7.07^b^	4.82 ± 0.59^g^	2.29 ± 0.15^a^
		9	14.18 ± 2.01^c^	0.70 ± 0.00^bcd^	3.64 ± 0.06^ab^	852.50 ± 3.53^b^	12.27 ± 1.94^abc^	2.22 ± 0.10^ab^
		13	12.38 ± 0.25^d^	0.70 ± 0.00^bcd^	3.92 ± 0.11^a^	852.50 ± 17.67^b^	8.01 ± 0.40^e^	1.84 ± 0.12^cd^
		17	11.23 ± 0.67^d^	0.75 ± 0.07^bc^	3.33 ± 0.32^bc^	855.00 ± 35.35^b^	7.76 ± 1.21^ef^	1.43 ± 0.15^e^
		21	12.43 ± 0.18^d^	0.70 ± 0.00^bc^	3.64 ± 0.29^ab^	925.00 ± 14.14^b^	9.78 ± 0.35^de^	1.14 ± 0.08^h^
		26	13.95 ± 0.49^cde^	0.65 ± 0.06^c^	3.32 ± 0.16^bc^	920.00 ± 21.21^b^	8.56 ± 0.57^e^	1.15 ± 0.02^h^
		31	14.08 ± 0.74^c^	0.75 ± 0.07^bc^	3.44 ± 0.18^bc^	887.50 ± 67.17^b^	11.92 ± 0.81^bc^	1.26 ± 0.08^gh^
	Storage	45	13.50 ± 0.49^ef^	0.60 ± 0.00^d^	3.37 ± 0.08^bc^	882.50 ± 17.68^b^	11.22 ± 1.15^cd^	1.50 ± 0.12^efg^
		60	11.32 ± 0.40^f^	0.75 ± 0700^bc^	3.25 ± 0.21^bc^	875.00 ± 7.07^b^	11.16 ± 1.03^cd^	1.32 ± 0.05^gh^
		75	12.40 ± 0.69^f^	0.80 ± 0.00^b^	3.44 ± 0.19^bc^	888.50 ± 44.54^b^	12.57 ± 0.01^abc^	1.59 ± 0.09^def^
		90	12.05 ± 0.43^f^	0.75 ± 0.07^bc^	3.32 ± 0.14^bc^	901.00 ± 41.01^b^	12.79 ± 0.32^abc^	1.67 ± 0.08^cde^
		120	12.05 ± 0.00^f^	0.95 ± 0.07^a^	3.30 ± 0.13^bc^	850.00 ± 42.43^b^	13.52 ± 0.70^ab^	1.72 ± 0.04^cde^
		150	12.55 ± 0.57^f^	1.00 ± 0.00^a^	3.21 ± 0.06^c^	915.00 ± 21.20^b^	14.24 ± 0.58^a^	1.57 ± 0.18d^efg^

Values are means of quadruplicates ± SD. The different letters (a–g) after value are significantly different (p < 0.05). MDI, the inner layers of MT-Daqu; MDO, the outer layers of MT-Daqu.

### Dynamics of culture-dependent microorganisms

**FIGURE 1:**
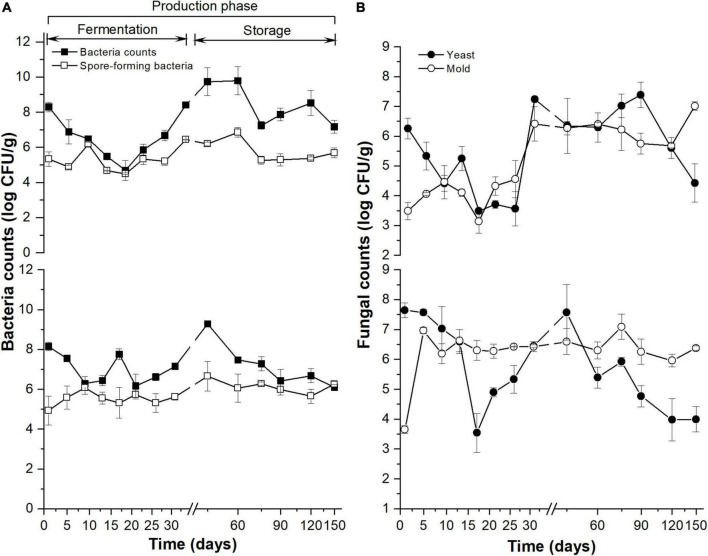
Dynamics of culture-dependent bacteria **(A)** and fungi **(B)** in MT-*Daqu* during entire production, including 31-day fermentation period and 4 months storage. The upper and lower *y*-axis indicate the microbial counts in MDI (the inner layers of MT-*Daqu*) and MDO (the outer layers of MT-*Daqu*), respectively.

The aerobic bacteria (Figure 1A, the upper y-coordinate) and yeast counts (Figure 1B, the upper y-coordinate) in MDI showed a similar pattern in the 31-day fermentation: reaching the minimum value on the 17th day. The aerobic bacteria in MDO (Figure 1A, the bottom of the *y*-axis) decreased rapidly in the first 9 days, then increased slowly to reach the maximum at 45 days, and subsequently decreased and stabilized at 90 days. The total yeast in MDO (Figure 1B, the bottom y-coordinate) decreased rapidly to reach the minimum value on the 17th day, then was restored to its original counts on the 45th day, and continued to decrease during storage. The total spore-forming bacteria counts in both parts of *Daqu* showed no obvious difference and similarly increased during the fermentation period and remained stable in the storage period (Figure 1A). In the fermentation period, the changes in mold in MDI (Figure 1B, the upper y-coordinate) were similar to those in spore-forming bacteria in MDI, whereas the mold in MDO increased rapidly in the first 5 days and remained stable thereafter (Figure 1B, the bottom of the *y*-axis).

**FIGURE 2:**
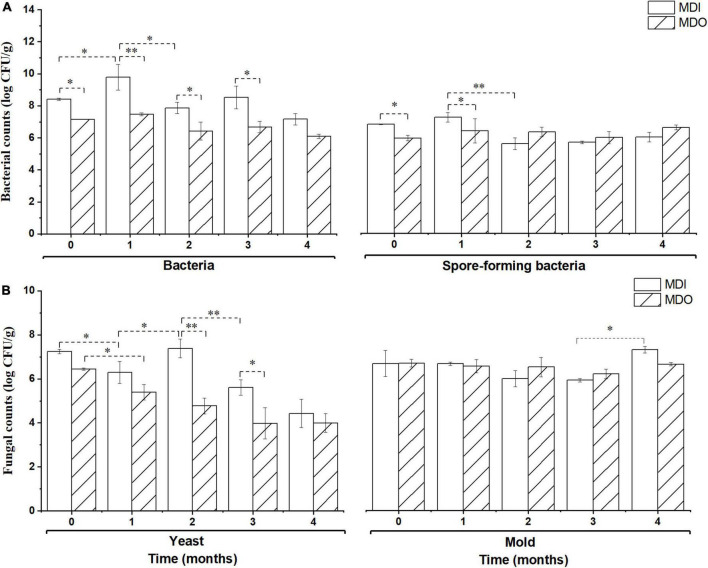
Comparison of the bacteria **(A)** and fungi **(B)** in the MDI and MDO samples during storage. Significant differences are noted by * (*p* < 0.05) and ^**^ (*p* < 0.01).

As shown in Figure 2, during storage, the microbial counts, except those of molds, in the MDI were significantly higher than those in the MDO (*p* < 0.05) in the first 2 months, whereas counts of the four groups of microbial in the MDI were close to those of MDO after 3 months of storage. Furthermore, all microbial counts in the MDO remained relatively constant after 1 month of storage.

### Composition of representative bacteria and fungi in mature medium-temperature-*Daqu*

A total of eighty-two bacterial isolates were obtained from the mature samples. Based on the analysis of the 16S rRNA gene sequence, 6 bacterial genera (15 species) were identified (Supplementary Table 1). Of these, *Bacillus* and *Staphylococcus* accounted for 70.7 and 13.4% of the bacterial community, respectively, at the genus level. A total of fifty fungal isolates were selected for identification by ITS region sequencing and 18S rRNA gene sequence analysis. A total of nine fungal species were identified, including *Wickerhamomyces anomalus* (12 strains), *Pichia jadinii* (8 strains), *Aspergillus awamori* (13 strains), *Aspergillus ustus* (4 strains), *Penicillium purpurogenum* (7 strains), *Penicillium sp.* (1 strain), *Alternaria alternata* (4 strains), and *Lichtheimia corymbifera* (1 strain) (Supplementary Table 2). *W. anomalus* and *A. awamori* mainly appeared in MDI, whereas other fungal species exhibited a similar distribution in both parts of MT-*Daqu*.

### Analysis of the microbial community of mature medium-temperature-*Daqu* using Illumina sequencing

**FIGURE 3:**
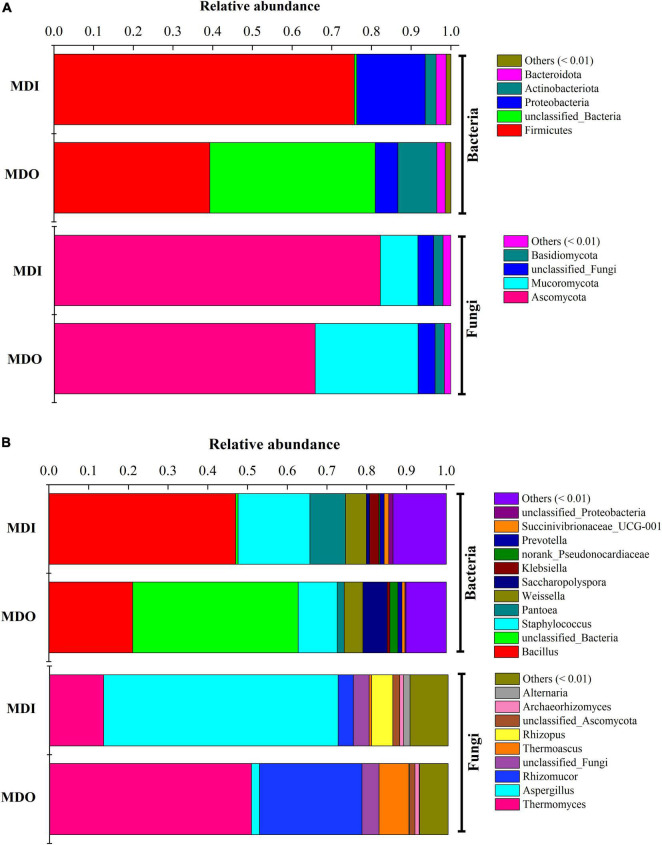
Microbial composition in different parts of the mature MT-*Daqu*. **(A)** Bacterial and fungal composition at the phylum level (more than 0.5%); **(B)** bacterial and fungal composition at the genus level (more than 0.5%). MDI, inner layers of MT-*Daqu*; MDO, outer layers of MT-*Daqu*.

Illumina sequencing of the bacterial 16S rRNA genes and fungal ITS gene generated a total of 283,896 and 520,350 effective sequences, respectively. After clustering all the sequences, 597-632 and 625-773 OTUs of observed species in the outer layer and inner layer of MT-*Daqu* were obtained, respectively. The community diversity of either bacteria or fungi in the MDI was greater than that in the MDO, whereas the number of bacterial OTUs (observed species) was less than that of fungal OTUs (Supplementary Table 3). At the phylum level (Figure 3A), the three dominant bacterial phyla were *Firmicutes* (75.68%), *Proteobacteria* (17.27%), and *Actinobacteria* (2.72%) in MDI. In MDO, *Firmicutes* and *Proteobacteria* were still dominant, but at lower percentages, e.g., only 39.26 and 5.74%, respectively. Moreover, the two dominant fungal phyla were *Ascomycota* and *Mucoromycota*, and the former was the predominant type, which accounted for 82.20 and 65.79% of the total sequences in MDI and MDO, respectively. At the genus level (Figure 3B), bacteria were dominated by *Bacillus*, *Staphylococcus*, *Pantoea*, *Weissella*, *Saccharopolyspora*, *Klebsiella*, and *Prevotella*. *Bacillus* and *Staphylococcus* were highly abundant (>9.83%) in the MDI and MDO samples, whereas *Bacillus* was relatively more abundant in MDI (47.02%) than in MDO (21.07%), and *Saccharopolyspora* was much less abundant in MDI (0.79%) than in MDO (6.11%). For fungi, *Thermomyces*, *Aspergillus*, *Rhizomucor*, *Thermoascus*, *Rhizopus*, *Archaeorhizomyces*, and *Alternaria* were the predominant genera in both parts of MT-*Daqu*. The abundances of *Aspergillus* and *Rhizopus* in MDI (58.88 and 5.34%, respectively) were higher than in MDO (20.05 and 0.14%). However, the abundances of *Thermomyces*, *Rhizomucor*, and *Thermoascus* in the MDI (13.56, 3.82, and 0.60%, respectively) were less than in MDO (50.67, 25.72, and 7.51%, respectively).

### Analysis of community structure in different parts of mature medium-temperature-*Daqu*

**FIGURE 4:**
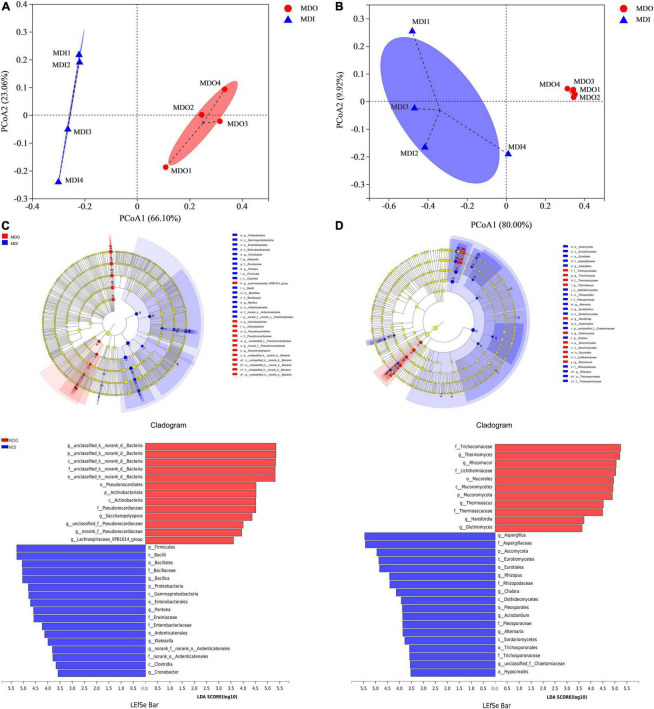
Principal coordinates analysis score plots of microbial β-diversity based on Bray–Curtis distance (*p* < 0.05) and LEfSe analysis of the MDI and MDO samples (LDA > 3.5, *p* < 0.05). Bacterial **(A,C)** and fungal **(B,D)** taxa showed significantly different abundances.

Principal coordinates analysis showed that the cumulative contribution of the first two principal coordinates was 89.16% for bacterial communities (Figure 4A) and 89.92% for fungal communities (Figure 4B). Moreover, the two PCoA plots showed that the samples of the same part of MT-*Daqu* clustered together, and PERMANOVA revealed that the differences were significant (*p* = 0.034 < 0.05). LEfSe indicated that as many as 30 bacteria (Figure 4C) and 29 fungi (Figure 4D) exhibited significant differences (*p* < 0.05) in different parts of MT-*Daqu* samples. *Bacillus*, *Klebsiella, Cronobacter*, and *Pantoea* were the most significantly enriched bacterial genera in MDI, whereas *Saccharopolyspora*, unclassified or no rank *Pseudonocardiaceae*, and unclassified *Bacteria* were enriched in MDO. A total of eighteen taxa and 11 taxa exerted significant effects (*p* < 0.05) on fungal community structure in the MDI and MDO samples, respectively. Furthermore, *Aspergillus*, *Alternaria*, and *Rhizopus* were enriched fungal genera in the MDI but *Thermomyces*, *Thermoascus*, and *Rhizomucor* were enriched in the MDO. At the genus level, the different types of samples contained a higher proportion of type-specific microorganisms.

### Analyses of volatile compounds in different parts of mature medium-temperature-*Daqu*

**FIGURE 5:**
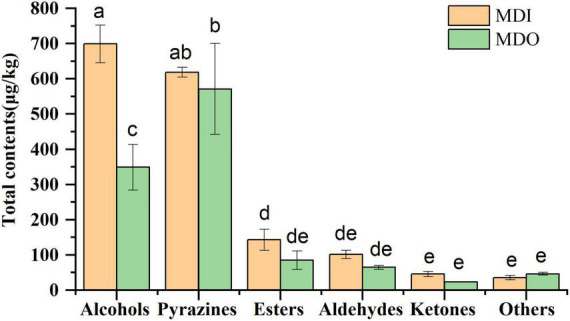
Relative abundance of six categories of volatile compounds in MDI and MDO samples of the mature MT-*Daqu*. Different letters indicate significant differences (*p* < 0.05).

A total of sixty-five volatile compounds, including 17 alcohols, 10 esters, 13 aldehydes, 7 ketones, 10 pyrazines, and 8 other compounds, were identified and semiquantified (Supplementary Table 4). Of these, isoamyl alcohol, hexanol, 2,3-butanediol, benzyl alcohol, phenylethyl alcohol, ethylhexanoate, ethylpalmitate, ethyllinoleate hexanal, benzaldehyde, geranylacetone, and tetramethylpyrazine were dominant in mature MT-*Daqu*. The contents of six species of volatile compounds are shown in Figure 5. The contents of alcohols and pyrazines were the highest in all samples, and only the alcohol content in the MDI (699.05 μg/kg) was significantly higher than that in the MDO (348.88 μg/kg). In the MDI sample, no significant difference was observed between the contents of alcohols and pyrazines, whereas in the MDO sample, the content of pyrazines at 590.96 μg/kg was the highest, which was 25 times higher than the lowest content of ketones (23.57 μg/kg).

## Discussion

The investigation of the culture-dependent microbial abundance and physicochemical properties of Daqu provided the first insight into the microbial composition and metabolic changes, which provides a basis for further omics analyses (Huang et al., 2020) and might be used to initially evaluate the maturity of *Daqu*. Despite the high workload and incomplete coverage, culture-dependent methods isolate cultivable strains that can be used as functional starters with contributions to the quality of ferment liquor and foods (Zheng et al., 2012; Lv et al., 2015; Yang et al., 2022). Considering bacteria, yeasts and molds as the three major types of microbiota in *Daqu* (Cai et al., 2021) and spore-forming bacteria, especially *Bacillus* as producers of enzymes and aroma compounds that contribute to *Daqu* flavor and quality (Yan et al., 2013b; He et al., 2019), two groups of bacteria, namely, aerobic bacteria and spore-forming bacteria and two groups of fungi, namely, yeast and mold, were analyzed using culture-dependent methods during MT-*Daqu* production. In the fermentation period, the counts of aerobic bacteria in MDI and yeast in both parts of MT-*Daqu* rapidly decreased to the minimum values at 17 days (Figures 1A,B) as the temperature increased to 60°C (Supplementary Figure 2) and then increased until the end of fermentation, consistent with previous findings (Li et al., 2016; Yan et al., 2019). This result might be attributed to the decrease in non-thermotolerant bacterial genera, such as lactic acid bacteria (LAB), and fungal genera, such as *Pichia*, which was restrained when the temperature was higher than 40°C (Li P. et al., 2015). The counts of spore-forming bacteria and molds in both parts of MT-*Daqu* increased rapidly in the first 9 days during the *Peijun* stage and then decreased slowly from 9 days to 17 days at the *Dinghuo* stage, thereafter increasing gradually or remaining stable until the end of fermentation. In the *Peijun* and *Dinghuo* stages, thermotolerant spore-forming bacteria, such as *Bacillus*, and filamentous fungi, such as *Aspergillus*, *Rhizopus*, *Rhizomucor*, and *Amylomyces*, survived and were commonly involved in solid-state fermentation with increasing temperature and decreasing moisture and increasing environmental relative humidity (Supplementary Figure 2; Li P. et al., 2015; Shanqimuge et al., 2015; Yan et al., 2019). This finding was confirmed by the changes in the total aerobic bacteria in MDI. The counts of total aerobic bacteria were essentially coincident with those of spore-forming bacteria from 9 days to 17 days, showing that the spore-forming bacteria are capable of producing endospores that are highly resistant to unfavorable environmental conditions of heat and desiccation, and thus, they were predominant among the total aerobic bacteria in this stage (Shanqimuge et al., 2015). Additionally, the cell counts of the four microbial groups in MDO were higher than those in MDI from 5 days to 21 days, which is fairly consistent with the results of previous studies (Zheng et al., 2012). The explanation for this observation may be that less access to water and air and a higher temperature MDI were not suitable for the growth of bacteria, except spore-forming bacteria, whereas the interface containing abundant air and nutrient substances easily formed in MDO, leading to the promotion of microbial growth (Li et al., 2013; Zhang Y. et al., 2021).

Consistent with previous studies, the cell counts of aerobic bacteria and yeasts in MDI and MDO samples were significantly different before and after the samples were stored for 3 months, and a similar difference was observed in the counts of spore-forming bacteria before 2 months (Li et al., 2013; Jin et al., 2019). These results supported the hypothesis that these environmental conditions promote three groups of microorganisms to propagate further in the inner layers. After at least 3 months of storage, the microbial counts in both parts of MT-*Daqu* were not significantly different (*p* > 0.05) because the changes in the environmental conditions, including temperature, moisture, and acidity, during storage, and drove the microorganisms in both parts of MT-*Daqu* to become similar and eventually form a stable system (Zhang Y. et al., 2021). Overall, culture-dependent microorganisms are stabilized after 3 months of storage.

The acidity of both parts quickly peaked in the early stage of fermentation, and the value of MDI was higher than that of MDO before 3 months (Table 1). Acidity was regarded as the most significant factor correlated with the composition of the microbial communities in the early stage of *Daqu* fermentation, and some *Lactobacillus* species were detected at higher percentages in the central part (Li et al., 2013), leading to the continuous synthesis of numerous organic acids (Shanqimuge et al., 2015). The liquefying activity and the saccharifying activity of *Daqu* are the two major contributors to liquefaction and saccharification in *Baijiu* production. Therefore, the two activities in different parts of *Daqu* showed similar changes throughout production (Table 1), and the two indices in MDI after fermenting 5 days and in MDO during production exhibited significantly positive correlations (Pearson correlation coefficient ρ = 0.58, *p* < 0.05 and ρ = 0.81, *p* < 0.01). The results might be attributed to the thermal stability of amylase and glucoamylase at high incubation temperatures of approximately 60°C (Li P. et al., 2015). Moreover, changes in both activities in the MDI and the MDO samples were similar to the counts of mold and spore-forming bacteria, respectively. A few studies have reported that some mold species, such as *Thermoascus*, *Aspergillus*, and *Rhizomucor*, and spore-forming bacterial species, such as *B. licheniformis*, *B. subtilis*, and *B. cereus*, which were identified as the core genera in *Daqu* production, produce various hydrolytic enzymes for starch liquefication and saccharification (Li Z. M. et al., 2015; Wang et al., 2018; Yan et al., 2019). Esterifying activity mainly contributes to the synthesis of esters that are the prominent flavor of *Baijiu* (Guan et al., 2021). In this study, along with the decrease in yeast counts, esterifying activity remained at a lower range until complete fermentation. Similar to acidity, the fermenting activity, especially in MDO, increased in the early fermentation period but did not change significantly in storage, mainly due to the higher counts of yeasts in the early phase (Fan et al., 2020).

Consistent with previous reports (Fan et al., 2020; Guan et al., 2021), we showed that storage for at least 3 months is necessary for MT-*Daqu* to reach a stable state in Figure 2. Moreover, we found that five main bacterial phyla, *Firmicutes*, *Proteobacteria*, *Actinobacteria*, *Cyanobacteria*, and *Bacteroidetes*, and two dominant fungal phyla, *Ascomycota* and *Mucoromycota*, were identified in both parts of MT-*Daqu*. These dominant microbial communities in MT-*Daqu* have been reported in other studies (Guan et al., 2021), and the same discovery was documented in both low-temperature and high-temperature *Daqu* (Li et al., 2013; Jin et al., 2019). *Bacillus*, *Klebsiella*, and *Pantoea* might represent potential biomarkers for MDI and *Saccharopolyspora* for MDO. The result that *Saccharopolyspora* were dominant outside of MT-*Daqu* is consistent with a previous study, and the study indicated that they were derived from the environment or raw materials to provide the initial microbiota (Zhang Y. et al., 2021). In addition, large amounts of *Bacillus*, *Staphylococcus*, and *Weissella* emerged in both samples. This finding was consistent with the analysis in terms of the predominance of *Bacillus* and *Staphylococcus* isolated and identified using culture-dependent methods (Supplementary Table 1). This study also showed that fungal communities in MT-*Daqu* were mainly comprised of *Aspergillus*, *Thermomyces*, *Rhizomucor*, *Thermoascus*, and *Rhizopus*, and *Aspergillus* and *Rhizopus* were dominant in MDI, whereas *Thermomyces*, *Rhizomucor*, and *Thermoascus* were predominant in MDO. The same composition of molds at the genus level in MT-*Daqu* was observed in liquor industries of South China, such as Sichuan and Jiangsu, which are the dominant regions producing strong-flavor *Baijiu* (Guan et al., 2021). Additionally, LEfSe revealed that the fungal genera *Aspergillus*, *Alternaria*, and *Rhizopus* were enriched in the MDI, whereas *Thermomyces*, *Thermoascus*, and *Rhizomucor* were enriched in the MDO. Unlike their abundances in high-temperature *Daqu*, *Byssochlamys*, and *Fusarium* were predominant in the inner and outer parts of high-temperature *Daqu*, respectively, and *Thermoascus* possessed an absolutely dominant position in both positions (Zhang Y. et al., 2021). In MT-*Daqu*, we did not detect *Byssochlamys* and only detected a low abundance of *Fusarium* (0.013%). These results may be due to the different production processes for different types of *Daqu* or different regional environments (Cai et al., 2021). Therefore, MT-*Daqu* has a specific fungal community structure. *Aspergillus*, *Alternaria*, and *Rhizopus* might be considered as biomarkers for MDI, whereas *Thermomyces*, *Thermoascus*, and *Rhizomucor* may be considered as biomarkers for MDO of MT-*Daqu* (Hu et al., 2021). The dominant fungal community comprised of molds was discovered in high-temperature and MT-*Daqu* (Hu et al., 2017; Wang et al., 2018), whereas the fungal composition with a high proportion of yeasts, such as *Wickerhamomyces*, *Saccharomycopsis*, and *Pichia*, was detected in low-temperature *Daqu* because of its fermentation at a lower temperature (Cai et al., 2021; Hu et al., 2021). Therefore, the special fungal composition of MT-*Daqu* is different from that of low-temperature *Daqu* but close to that of high-temperature *Daqu*, which potentially explains why the strong-flavor *Daqu* has its own unique flavor and physiological–biochemical characteristics.

Flavors in *Daqu* play an important role in the flavor classification of Chinese *Baijiu* (Zhang et al., 2011; Wang et al., 2020). In this study, isoamyl alcohol, hexanol, 2,3- butanediol, benzyl alcohol, phenylethyl alcohol, ethylhexanoate, ethylpalmitate, ethyllinoleate, hexanal, benzaldehyde, geranylacetone, and tetramethylpyrazine were detected as the predominant flavors in mature MT-*Daqu* (Supplementary Table 4), consistent with the findings of *Luzhou*-*Daqu* (Sichuan, China) (Zhang et al., 2011; He et al., 2019) and *Yingjia*-*Daqu* (Anhui, China) (Yan et al., 2019). Of these compounds, tetramethylpyrazine was present the highest relative abundance in both parts of MT-*Daqu*, followed by phenylethyl alcohol and benzaldehyde. Pyrazines, which are nitrogen-containing compounds, are the important volatile compounds of Chinese *Baijiu* that provide baked, roasted, and nutty notes (Fan and Qian, 2006) and are reported to be the key active compounds of Jiang-flavor *Baijiu* (Jin et al., 2019). A total of ten pyrazines were also identified in our samples, five of which were detected in “*Wuliangye*” and “*Jiannanchun*” strong-flavored *Baijiu* (Fan and Qian, 2006). He et al. (2019) reported that 10 pyrazines were identified in *Luzhoulaojiao*-*Daqu*, and the relatively high content of tetramethylpyrazine was consistent with the high abundance of *Bacillus* in fortified MT-*Daqu* inoculation with *Bacillus velezensis* and *Bacillus subtilis* (He et al., 2019). According to Jin et al. (2019), *Bacillales*, *Thermoactinomyces*, and *Aspergillus* are the principal contributors to pyrazines (Jin et al., 2019). We found that alcohols were also the most dominant flavor compounds and total alcohols were detected at the highest abundance in MDI (Figure 5). Alcohols, as important precursors of acids and esters, comprise the largest group of compounds, and most of them have been detected in Chinese liquor, which often impart fruity, floral, green, and alcoholic odors and contribute to the flavor of Chinese liquor (Zhang et al., 2011). Phenethyl alcohol, imparting floral components, such as rose and fruity notes, was the main constituent in alcohols in MT-*Daqu* (Yang et al., 2021) and was the second most abundant compound in this study. In addition to phenethyl alcohol, isoamyl alcohol, hexanol, and S-2,3-butanediol were present at high contents in all samples, and their contents in MDI were higher than those in MDO. These higher alcohols might be formed from amino acid catabolism *via* the Ehrlich metabolic pathway or from lipid oxidation by some microbial strains, such as *Enterobacter*, *Acinetobacter*, *Aspergillus*, *Rhizopus*, and *Alternaria* (Zhang et al., 2011; Tang Q. X. et al., 2019; Yang et al., 2022). In addition, geranylacetone, which was present at an intermediate content, was first identified in MT-*Daqu*, and its function and source in *Daqu* are still unknown and require further exploration.

## Conclusion

This study provided a comprehensive understanding of the microbial community and physicochemical properties inside and outside the MT-*Daqu* used for strong-flavor *Baijiu* production from the fermentation period to the mature period. Both culture-dependent and culture-independent methods provided a view of changes in the microbial community during production and discrepancies in the different parts of the MT-*Daqu*. The physicochemical property indices in both parts of MT-*Daqu* shifted from the fermentation period to a relatively stable range after storage for 3 months, and a difference between the inner layers and the outer layers was also observed. The findings provide theoretical evidence for industrial process control and evaluating the mature period of MT-*Daqu*. This study also investigated the predominant bacterial and fungal composition and volatile compounds inside and outside the mature MT-*Daqu*. The results will assist in quality judgments of the mature MT-*Daqu* and identifying the functional microbial sources to apply in *Baijiu* brewing.

## Data availability statement

The original contributions presented in the study are included in the article/Supplementary material, further inquiries can be directed to the corresponding author.

## Author contributions

XH conducted all the experiments and wrote the initial manuscript. RL and MH conceived and designed the experiments. ZS and JW revised and proofread the manuscript. XS and RX analyzed the experimental data. XL and CP contacted the liquor manufacturer and participated in the partial production experiment. All authors read and approved the manuscript.
