# Supplementary material for: Comparative analysis of the microbiotas and physicochemical properties inside and outside medium-temperature Daqu during the fermentation and storage
Source: Front Microbiol. 2022 Jul 27;13:934696. doi: 10.3389/fmicb.2022.934696 (PMC9363831; doi:10.3389/fmicb.2022.934696)
Supplement: Supplementary file 1 [file Data_Sheet_1.PDF]

# *Supplementary Material*

## 1 Supplementary Figures and Tables

### 1.1 Supplementary Figure legends

**Supplementary Figure 1** The production workflow of MT-*Daqu* by traditional method.

**Supplementary Figure 2** Temperature and humidity changes in environment during 31 days fermentation.

**Supplementary Figure 3** Rarefaction curves of MT-*Daqu* samples., the sequencing depth of the bacterial 16S rRNA genes (A) and the fungal ITS gene (B).

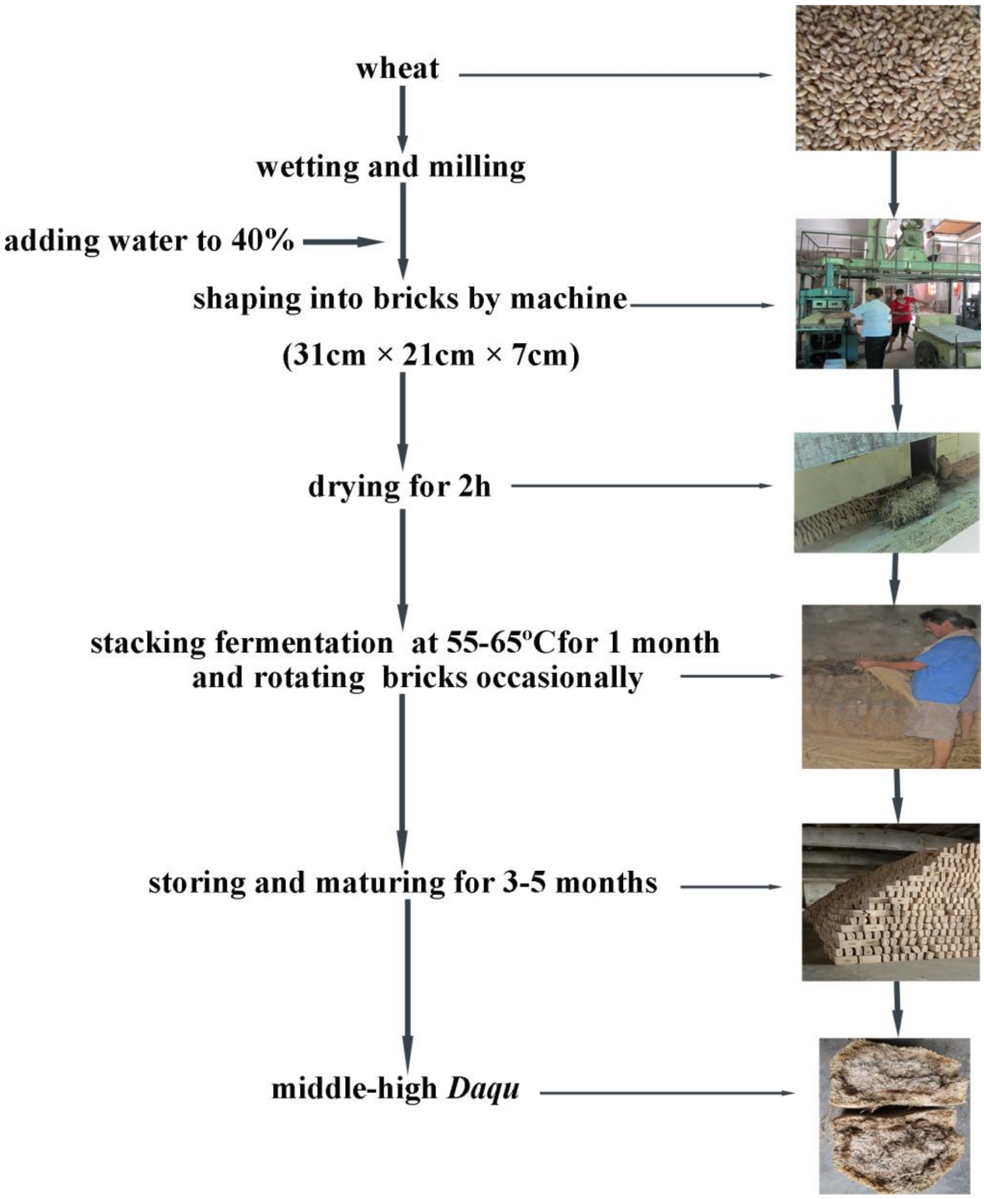


**Supplementary Figure 1** The production workflow of MT-*Daqu* by traditional method

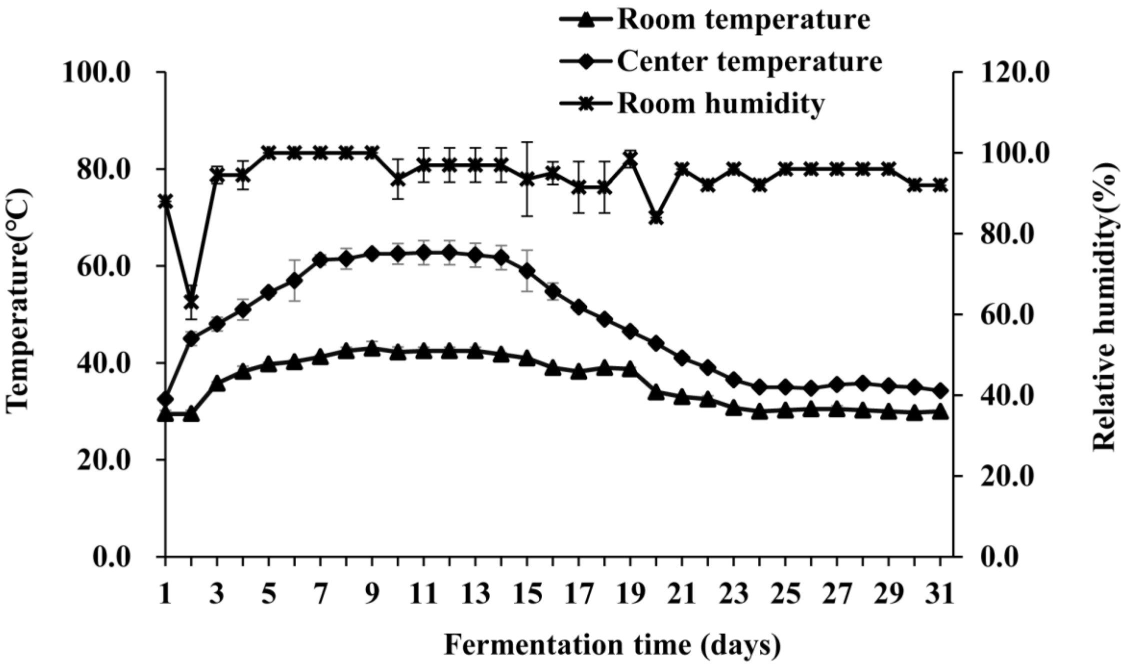


**Supplementary Figure 2** Temperature and humidity changes in environment during 31 days fermentation

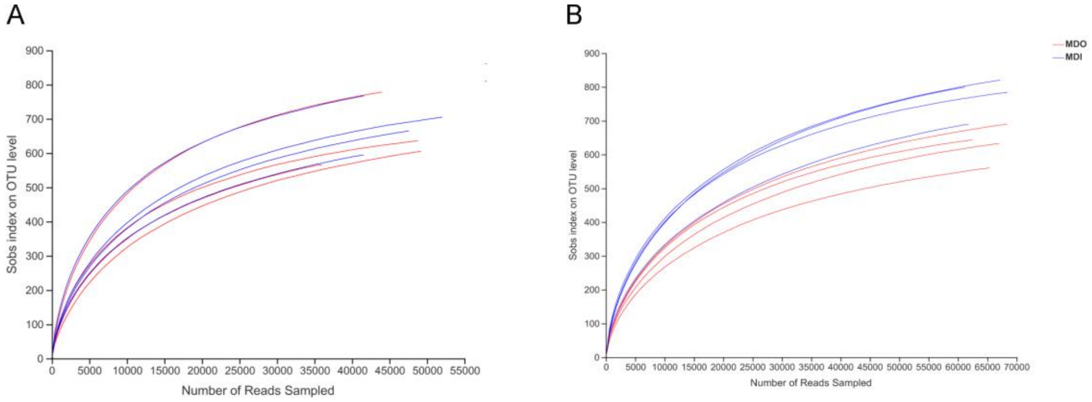


**Supplementary Figure 3** Rarefaction curves of MT-*Daqu* samples. The sequencing depth of the bacterial 16S rRNA genes (A) and the fungal ITS gene (B)

## 1.2 Supplementary Tables

**Supplementary Table 1** Identification of culture-dependent bacteria species by BLAST and distribution in the samples.

**Supplementary Table 2** Identification of culture-dependent fungal species by BLAST and distribution in the samples.

**Supplementary Table 3** Sequencing results and the alpha diversity analysis of different part of mature MT-*Daqu*.

**Supplementary Table 4** Identification and quantity of volatile compounds in different part of mature MT-*Daqu*.

**Supplementary Table 1** Identification of culture-dependent bacteria species by BLAST and distribution in the samples

| Species | Genus name | Accession No. | Identity (%) | Approximate sequences accession No. | MDI | MDO |
|---|---|---|---|---|---|---|
| *Bacillus licheniformis* | *Bacillus* | MN493766 | 100 | EU000058 | 11 | 6 |
| *Bacillus subtilis* | | MN493719 | 100 | DQ452509 | 8 | 6 |
| *Bacillus safensis* | | MN493773 | 100 | AY167880 | 6 | 4 |
| *Bacillus velezensis* | | OM074003 | 99 | CP041192.1 | 5 | 3 |
| *Bacillus altitudinis* | | OM074001 | 100 | NR_042337.1 | 2 | 1 |
| *Bacillus amyloliquefaciens* | | MN493771 | 100 | EU304965 | 2 | 2 |
| *Bacillus sonorensis* | | OM074006 | 100 | NR_113993.1 | 0 | 1 |
| *Bacillus sp.* | | MN493778 | 100 | AY462213 | 1 | 0 |
| *Staphylococcus gallinarum* | *Staphylococcus* | OM074005 | 100 | NR_036903.1 | 6 | 3 |
| *Staphylococcus saprophyticus subsp.* | | OM073987 | 99 | NR_036903.1 | 1 | 0 |
| *Staphylococcus arlettae* | | OM074002 | 99 | NR_024664.1 | 1 | 0 |
| *Thermoactinomyces vulgaris* | *Thermoactinomyces* | MN493782 | 99 | AF138732 | 4 | 1 |
| *Micrococcus sp.* | *Micrococcus* | MN493768 | 100 | EU182882 | 1 | 3 |
| *Pediococcus pentosaceus* | *Pediococcus* | OM085667 | 99 | NR_042058.1 | 2 | 1 |
| *Exiguobacterium sp.* | *Exiguobacterium* | MN493784 | 99 | AY205564 | 1 | 0 |

**Supplementary Table 2** Identification of culture-dependent fungal species by BLAST and distribution in the samples

| Species | Genus name | Accession No. | Identity (%) | Approximate sequences accession No. | MDI | MDO |
|---|---|---|---|---|---|---|
| *Wickerhamomyces anomalus* | *Wickerhamomyces* | MN514248 | 99 | KT175180.1 | 5 | 7 |
| *Pichia jadinii* | *Pichia* | MN498028 | 99 | FJ797687.1 | 6 | 2 |
| *Aspergillus awamori* | *Aspergillus* | MN514189 | 99 | HQ393870.1 | 10 | 3 |
| *Aspergillus ustus* | | MN514188 | 99 | GQ856237.1 | 3 | 1 |
| *Penicillium purpurogenum* | *Penicillium* | MN514190 | 100 | KC143068.1 | 4 | 3 |
| *Penicillium sp.* | | MN514180 | 99 | JX134614.1 | 1 | 0 |
| *Alternaria lternate* | *Alternarial* | MN514179 | 100 | HM165489.1 | 3 | 1 |
| *Lichtheimia corymbifera* | *Lichtheimia* | MN514181 | 99 | HM590658.1 | 1 | 0 |

**Supplementary Table 3** Sequencing results and the alpha diversity analysis of different part of mature MT-*Daqu*

| Genes | Sample | Raw tags | Clean tags | Nochime | Length(bp) | Observed species | Coverage | Chao | Ace | Shannon | Simpson |
|---|---|---|---|---|---|---|---|---|---|---|---|
| 16S rRNA | MDI | 314746 | 259779 | 194753 | 426 | 625 | 0.995 | 756 | 766 | 2.63 | 0.23 |
| | MDO | 299067 | 238469 | 185303 | 414 | 597 | 0.995 | 728 | 733 | 2.42 | 0.24 |
| ITS | MDI | 383774 | 65363 | 261451 | 255 | 773 | 0.997 | 883 | 916 | 2.72 | 0.18 |
| | MDO | 423831 | 66382 | 265529 | 266 | 632 | 0.997 | 741 | 756 | 1.92 | 0.33 |

**Supplementary Table 4** Identification and quantity of volatile compounds in different part of mature MT-*Daqu*

| Serial number | Number | Retention time (min) | Volatile compounds | Identification | Contents of volatile compounds (μg/kg) | |
|---|---|---|---|---|---|---|
| | | | | | MDI | MDO |
| | **Alcohols** | | | | | |
| 1 | A1 | 2.55 | 2-ethyl-2-Cyclobutanol | | 4.36±0.29 | nd |
| 2 | A2 | 5.30 | Butanol | MS | 4.59±0.06 | nd |
| 3 | A3 | 7.93 | Isoamyl alcohol | MS | 46.06±6.31 | 31.81±6.11 |
| 4 | A4 | 9.64 | Pentanol | MS | 4.52±6.40 | 7.08±1.62 |
| 5 | A5 | 12.30 | Prenol | MS | 17.83±0.04 | 17.51±3.59 |
| 6 | A6 | 13.43 | Hexanol | MS | 51.54±10.89 | 34.42±6.57 |
| 7 | A7 | 16.88 | 1-Heptanol | MS | 6.23±1.06 | 4.75±0.10 |
| 8 | A8 | 18.01 | 2-Ethyl-1-hexanol | MS | 12.41±5.61 | 8.30±1.32 |
| 9 | A9 | 19.53 | R-2,3-Butanediol | MS | 47.99±31.88 | 69.79±18.41 |
| 10 | A10 | 20.68 | S-2,3-Butanediol | MS | 46.05±0.38 | 25.14±6.74 |
| 11 | A11 | 21.81 | 2-Decen-1-ol | MS | nd | 1.13±0.60 |
| 12 | A12 | 22.42 | Cyclohexanol, 1-methyl-4-1-methylethyl | MS | 17.33±4.76 | 13.01±0.19 |
| 13 | A13 | 26.35 | A,a-dimethyl-Benzenemethanol | MS | 8.88±4.96 | 5.36±0.66 |
| 14 | A14 | 29.12 | Benzyl alcohol | MS | 126.65±23.82 | 32.79±3.81 |

| | | | | | | |
|---|---|---|---|---|---|---|
| 15 | A15 | 29.79 | Phenylethyl alcohol | MS | 307.60±6.02 | 92.55±15.02 |
| 16 | A16 | 31.89 | 2,5-dimethyl-4-Hexen-3-ol | MS | nd | 4.01±0.44 |
| 17 | A17 | 34.92 | ß-Acorenol | MS | nd | 1.23±0.74 |
| | **Esters** | | | | | |
| 18 | E1 | 0.48 | Ethylbutyrate | MS | 2.70±0.62 | nd |
| 19 | E2 | 8.96 | Ethylhexanoate | MS | 69.41±10.57 | 33.42±7.92 |
| 20 | E3 | 16.17 | Ethyloctanoate | MS | 4.26±1.44 | 3.42±1.07 |
| 21 | E4 | 21.33 | Stearic acid, 3-(octadecyloxy)propyl ester | MS | 0.00 | 2.39±0.45 |
| 22 | E5 | 26.81 | Cyclopentyl benzoate | MS | 3.05±1.55 | 4.53±0.94 |
| 23 | E6 | 35.11 | Methyl 2-aminobenzoate | MS | 2.60±1.05 | 0.00 |
| 24 | E7 | 35.28 | Ethylpalmitate | MS | 25.51±3.31 | 16.32±4.72 |
| 25 | E8 | 35.52 | E-11-Hexadecenoic acid, ethyl ester | MS | 2.97±1.51 | nd |
| 26 | E9 | 38.25 | Ethyl 9-octadecenoate | MS | 10.95±4.70 | 8.85±2.55 |
| 27 | E10 | 38.98 | Ethyl linoleate | MS | 21.65±4.23 | 16.17±5.35 |
| | **Aldehydes** | | | | | |
| 28 | AL1 | 2.33 | Hexanal | MS | 25.11±3.48 | 20.70±4.94 |
| 29 | AL2 | 7.57 | 3-methyl-2-Butenal | MS | 0.80±1.14 | nd |
| 30 | AL3 | 11.06 | Undecanal | MS | nd | 1.10±0.55 |

| | | | | | | |
|---|---|---|---|---|---|---|
| 31 | AL4 | 11.08 | Octanal | MS | nd | 1.04±0.47 |
| 32 | AL5 | 14.74 | Nonanal | MS | 4.79±2.04 | 4.14±1.01 |
| 33 | AL6 | 16.50 | E-14-Hexadecenal | MS | nd | 2.48±0.92 |
| 34 | AL7 | 18.19 | Decanal | MS | 15.00±0.11 | 8.45±0.01 |
| 35 | AL8 | 18.86 | Benzaldehyde | MS | 45.25±2.76 | 16.87±2.02 |
| 36 | AL9 | 19.25 | 2-Nonenal | MS | 3.94±0.06 | 5.44±0.67 |
| 37 | AL10 | 22.08 | 13-Tetradecenal | MS | 2.96±0.09 | nd |
| 38 | AL11 | 22.51 | Benzeneacetaldehyde | MS | 3.41±0.78 | 0.93±0.01 |
| 39 | AL12 | 24.40 | 2,4-Nonadienal | MS | nd | 0.91±1.29 |
| 40 | AL13 | 26.43 | 2,4-Decadienal | MS | nd | 2.69±0.36 |
| | **ketones** | | | | | |
| 41 | K1 | 5.52 | Tetrahydro-6,6-dimethyl-2H-Pyran-2-one | MS | nd | 1.24±0.75 |
| 42 | K2 | 6.31 | 8-Hydroxy-2-octanone | MS | 3.50±0.59 | nd |
| 43 | K3 | 6.91 | 2-Heptanone | MS | nd | 1.34±1.90 |
| 44 | K4 | 10.83 | 2-Octanone | MS | 15.34±1.53 | 4.50±0.10 |
| 45 | K5 | 22.66 | Acetophenone | MS | 9.08±1.38 | 4.75±0.31 |
| 46 | K6 | 24.92 | 4-Methyl-2(5H)-furanone | MS | 1.60±0.26 | nd |
| 47 | k7 | 28.62 | Geranylacetone | MS | 16.12±0.51 | 11.74±0.31 |
| | **Pyrazines** | | | | | |

| | | | | | | |
|---|---|---|---|---|---|---|
| 48 | P1 | 9.93 | methyl-2-Pyrazine | MS | 2.92±0.62 | nd |
| 49 | P2 | 11.97 | 2,5-dimethyl-Pyrazine | MS | 5.89±1.89 | 3.57±0.69 |
| 50 | P3 | 12.21 | 2,6-dimethyl-Pyrazine | MS | 3.50±0.89 | 2.59±0.50 |
| 51 | P4 | 12.83 | 2,3-dimethyl-Pyrazine | MS | 15.33±1.95 | 9.90±0.30 |
| 52 | P5 | 16.87 | 2-ethyl-3,5-dimethyl-2-Pyrazine | MS | 11.87±5.73 | 15.31±2.19 |
| 53 | P6 | 17.17 | Tetramethylpyrazine | MS | 555.57±14.14 | 519.22±122.39 |
| 54 | P7 | 17.32 | 2,3,5-Trimethyl-6-butylpyrazine | MS | 7.30±1.05 | 4.98±0.30 |
| 55 | P8 | 17.75 | 2-ethenyl-6-methyl-Pyrazine | MS | 2.00±0.82 | 2.06±0.37 |
| 56 | P9 | 18.47 | 2,3,5-Trimethyl-6-ethylpyrazine | MS | 6.083±1.71 | 6.50±1.20 |
| 57 | P10 | 20.27 | 6-Methyl-1,2,4-triazolo[4,3-b]pyridazine | MS | 7.88±0.14 | 6.82±1.45 |
| | **Acids and Others** | | | | | |
| 58 | AO1 | 1.31 | 4-Amino-1,5-pentandioic acid | MS | nd | 2.03±0.05 |
| 59 | AO2 | 23.64 | 3-methyl-Butanoic acid | MS | nd | 2.12±0.11 |
| 60 | AO3 | 38.84 | Hexadecanoic acid | MS | 1.71±0.42 | 2.32±3.28 |
| 61 | AO4 | 22.00 | Gamma Butyrolactone | MS | nd | 2.11±0.01 |
| 62 | AO5 | 22.08 | 1-phenyl-2-(1H-1,2,4-triazol-3-ylthio)-Ethanone | MS | 2.86±1.05 | nd |
| 63 | AO6 | 25.53 | 3-Hydroxymethylene-1,7,7-trimethylbicyclo[2.2.1]heptan-2-one | MS | 4.97±2.48 | 3.24±0.18 |
| 64 | AO7 | 33.19 | 2,5-Bisisobutylthiophene | MS | 16.45±3.53 | 28.52±6.65 |

| | | | | | | |
|---|---|---|---|---|---|---|
| 65 | AO8 | 34.55 | 2-Methoxy-4-vinylphenol | MS | 9.56±1.73 | 6.02±0.55 |
